# Pyrroloquinoline Quinone Mitigates Testicular Injury and Reduces Oxidative Stress, Mitochondrial Dysfunction, and Apoptosis in Rats with Testicular Ischemia–Reperfusion Injury

**DOI:** 10.3390/antiox14111312

**Published:** 2025-10-31

**Authors:** Syuan-Hao Syu, Chao-Yuan Chang, Hung-Jen Shih, Chun-Jen Huang

**Affiliations:** 1 Graduate Institute of Clinical Medicine, College of Medicine, Taipei Medical University, Taipei 110, Taiwan; 100454@w.tmu.edu.tw (S.-H.S.), 110234@w.tmu.edu.tw (C.-Y.C.); 2 Department of Urology, School of Medicine, College of Medicine, Taipei Medical University, Taipei 110, Taiwan; 3 Department of Urology, Wan Fang Hospital, Taipei Medical University, Taipei 116, Taiwan; 4 Department of Medical Research, Wan Fang Hospital, Taipei Medical University, Taipei 116, Taiwan; 5 Integrative Research Center for Critical Care, Wan Fang Hospital, Taipei Medical University, Taipei 116, Taiwan; 6 Division of Urology, Department of Surgery, Changhua Christian Hospital, Changhua 500, Taiwan; 7 Department of Post-Baccalaureate Medicine, College of Medicine, National Chung Hsing University, Taichung 402, Taiwan; 8 Department of Anesthesiology, School of Medicine, College of Medicine, Taipei Medical University, Taipei 110, Taiwan; 9 Department of Anesthesiology, Wan Fang Hospital, Taipei Medical University, Taipei 116, Taiwan

**Keywords:** pyrroloquinoline quinone, testicular torsion–detorsion, ischemia–reperfusion injury, oxidative phosphorylation, oxidative stress, mitochondrial dysfunction, apoptosis

## Abstract

Testicular torsion–detorsion (T/D) induces ischemia–reperfusion injury, leading to mitochondrial dysfunction, oxidative stress, apoptosis, and spermatogenic impairment. Pyrroloquinoline quinone (PQQ), a redox cofactor with mitochondrial-protective, antioxidant, and anti-apoptotic properties, was evaluated for its therapeutic potential in a rat T/D model. Young adult male Sprague-Dawley rats underwent 720° spermatic cord rotation for 2 h followed by detorsion and were assigned to T/D or T/D + PQQ groups, with sham-operated controls run in parallel. PQQ (400 mg/kg body weight) was administered orally once daily for 4 weeks. T/D resulted in severe disruption of testicular architecture, disorganization of seminiferous epithelium, reduced sperm count and testis-to-body weight ratio, increased hypoxia-inducible factor-1α and malondialdehyde, decreased superoxide dismutase 2, impaired oxidative phosphorylation (OXPHOS), and enhanced apoptosis. Notably, PQQ treatment significantly preserved testicular structure, improved sperm counts, reduced oxidative stress, restored OXPHOS, and suppressed apoptosis (all *p* < 0.05. T/D + PQQ vs. T/D). These findings indicate that PQQ protects against T/D-induced testicular injury. The underlying mechanisms may involve the attenuation of oxidative stress, the preservation of mitochondrial function, and the limitation of apoptosis, supporting its potential as a therapeutic strategy for testicular IRI.

## 1. Introduction

Testicular torsion is a urological emergency characterized by acute scrotal pain resulting from twisting of the spermatic cord which obstructs testicular blood flow [1]. Without prompt surgical detorsion, ischemia rapidly progresses to irreversible testicular necrosis [1]. Paradoxically, detorsion itself can precipitate reperfusion injury in previously ischemic tissue, making testicular torsion–detorsion (T/D) a well-established experimental model for studying ischemia–reperfusion injury [1,2,3,4]. This model faithfully reproduces the hemodynamic, biochemical, and inflammatory cascades triggered by abrupt restoration of blood flow—most notably the excessive generation of reactive oxygen species (ROS), leukocyte infiltration, and oxidative damage to the seminiferous epithelium and germ cells.

Clinically, even after successful surgical detorsion, many patients experience residual testicular dysfunction, subfertility, or atrophy due to reperfusion-induced oxidative stress and inflammation [5]. Because spermatogenic integrity and parenchymal preservation are critical to male fertility [6], interventions that can attenuate oxidative injury and apoptosis after detorsion are urgently needed. The rat T/D model provides a reproducible and pathophysiologically relevant system to evaluate such strategies, allowing detailed assessment of histological, biochemical, and molecular endpoints related to testicular recovery.

In T/D-induced ischemia–reperfusion injury, profound hypoxia upregulates hypoxia-inducible factor-1α (HIF-1α), a master regulator of oxygen-deprivation responses [7]. Reperfusion produces a burst of ROS that overwhelms antioxidant defenses, causing lipid peroxidation—marked by elevated malondialdehyde (MDA)—and reducing superoxide dismutase 2 (SOD-2), a key ROS-detoxifying enzyme [8]. Sustained oxidative stress disrupts mitochondrial structure and oxidative phosphorylation (OXPHOS), activates intrinsic apoptotic pathways, and culminates in executioner caspase activation, particularly cleaved caspase-3, as shown in rodent T/D models [9,10,11,12]. HIF-1α, MDA, SOD2, defective OXPHOS, and cleaved caspase-3 function as pivotal molecular drivers of hypoxia responses, oxidative injury, impaired antioxidant defense, mitochondrial dysfunction, and apoptosis, respectively, thereby orchestrating the cascade of cellular damage in T/D-induced ischemia–reperfusion injury and providing rational targets for therapeutic intervention.

Our previous studies have demonstrated that pharmacologic agents such as simvastatin, hemin, and FTY720 can alleviate T/D-induced ischemia–reperfusion injury in rats by enhancing antioxidant defenses, suppressing inflammation, and reducing apoptosis [13,14,15]. Notably, germ cell apoptosis caused by T/D was also attenuated by FTY720 [4]. However, these treatments primarily addressed the acute phase of injury (within 24–72 h) and required systemic administration via intraperitoneal injection. Therapeutic strategies effective during the subacute and recovery phases—when spermatogenic remodeling occurs—remain largely unexplored. From a translational standpoint, orally deliverable compounds capable of sustained antioxidant and mitochondrial protection would offer greater feasibility and patient compliance.

**Figure 1:**
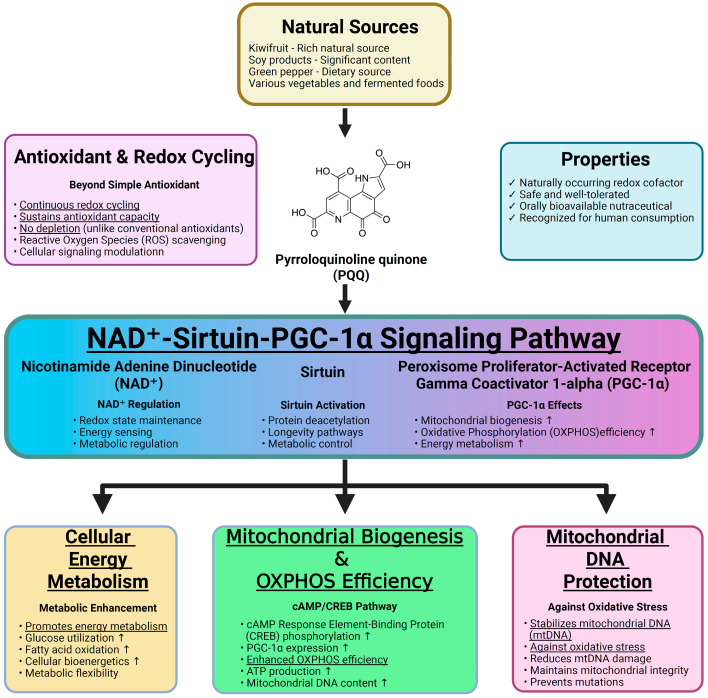
Effects and mechanisms of pyrroloquinoline quinone (PQQ).

Pyrroloquinoline quinone (PQQ) is a naturally occurring redox cofactor first isolated from bacterial dehydrogenases and later identified in various foods such as kiwifruit, soy, green peppers, and fermented products [16]. It is recognized as a nutritionally important compound with excellent oral bioavailability and safety in both animals and humans, showing no toxicity even at high supplemental doses [16,17,18]. Unlike conventional antioxidants that scavenge ROS in a single reaction, PQQ undergoes continuous redox cycling, thereby maintaining long-lasting antioxidant capacity. Mechanistically, PQQ promotes mitochondrial biogenesis and enhances OXPHOS efficiency via the NAD^+^–sirtuin–peroxisome proliferator-activated receptor gamma coactivator 1-α (PGC-1α) pathway, improving energy metabolism and stabilizing mitochondrial DNA (mtDNA) [16,17,18,19]. These pleiotropic effects make PQQ a promising candidate for targeting the oxidative and mitochondrial components of T/D-induced IRI. The principal mechanisms and effects of PQQ are summarized in Figure 1.

Oral PQQ supplementation has been shown to reduce ROS levels and improve reproductive performance in mice [20]. Moreover, PQQ’s ability to cross cellular and mitochondrial membranes allows it to directly mitigate mitochondrial oxidative injury—a central pathological event in T/D-induced ischemia–reperfusion injury [11,16,17,18]. Despite its promising pharmacological profile and safety record, the protective role of PQQ in testicular T/D-induced ischemia–reperfusion injury has not been systematically investigated. Addressing this gap, in this study we aimed to investigate the long-term therapeutic effects of daily oral PQQ supplementation for four consecutive weeks after T/D, a critical period reflecting the subacute and recovery phases of spermatogenic remodeling. We hypothesized that prolonged oral PQQ administration would (1) attenuate testicular injury and preserve spermatogenesis in a rat T/D model and (2) exert these effects by reducing oxidative stress, maintaining OXPHOS function, and suppressing apoptosis. The findings from this study may provide mechanistic insight into mitochondria-targeted antioxidant therapy and support the development of a clinically feasible, orally administrable approach to preserve testicular function following surgical detorsion and mitigate T/D-induced ischemia–reperfusion injury.

## 2. Materials and Methods

### 2.1. Animals

Adult male Sprague-Dawley rats (8–9 weeks old, weighing 200–250 g; BioLASCO Taiwan Co., Taipei, Taiwan) were used. Rats were housed under temperature- and light-controlled conditions (12 h light/12 h dark cycle) with free access to water and standard chow. All procedures were approved by the Institutional Animal Care and Use Committee of Taipei Medical University (LAC2023-0438) and conducted in accordance with the US National Institutes of Health (NIH) guidelines for the care and use of laboratory animals to ensure compliance with international animal welfare standards.

### 2.2. T/D and Sham Surgery

T/D surgery was conducted as previously described [4]. Under anesthesia induced by intraperitoneal injection of Zoletil (40 mg/kg; Virbac Korea Co., Ltd., Seoul, Republic of Korea) and xylazine (10 mg/kg; Sigma-Aldrich, St. Louis, MO, USA), rats were placed in the supine position on a heating pad. Through a mid-scrotal vertical incision, the left testis was rotated 720° counterclockwise and secured to the scrotum with a silk suture through the tunica albuginea for 2 h, then detorsed and returned to its anatomical position. To serve as the control, sham-operated rats underwent identical exposure, gentle manipulation without torsion, and closure using the same technique.

### 2.3. Study Design

Rats were randomly assigned to four groups (*n* = 6 per group): (1) Sham (sham surgery without treatment), (2) PQQ (sham surgery with PQQ treatment), (3) T/D (T/D surgery without treatment), and (4) T/D + PQQ (T/D surgery with PQQ treatment). Rats in the Sham + PQQ and T/D + PQQ groups received PQQ (400 mg/kg body weight in 300 μL normal saline) by oral gavage once daily for 4 consecutive weeks, starting immediately after wound closure. Rats in the Sham and T/D groups received an equal volume of normal saline by gavage following the same schedule.

The dose of PQQ was determined based on a previous study reporting that daily oral administration of PQQ at 100, 200, and 400 mg/kg body weight for 13 weeks produced no detectable toxicological abnormalities in rats, establishing 400 mg/kg body weight/day as the no-observed-adverse-effect level [21]. Moreover, this dose has been shown to confer significant antioxidant effects [22].

### 2.4. Testicular Harvesting

At 28 days after detorsion in the T/D groups, or at the corresponding time point in the Sham groups, all surviving rats from each group were euthanized by decapitation under deep anesthesia. The bilateral testes were collected and epididymis was freshly removed for the sperm counting assay. Samples were kept at 37 °C until use or, if not processed immediately, stored in phosphate-buffered saline (PBS; Sigma-Aldrich) at room temperature. The testes were longitudinally divided into two halves. One half was snap-frozen in liquid nitrogen, while the other half was immersed in Bouin’s solution (Sigma-Aldrich, St. Louis, MO, USA).

### 2.5. Sperm Counting Assay

Sperm counting assay was performed as previously reported [4]. In brief, both cauda epididymides were removed and weighed. Each epididymis was minced in 1 mL of pre-warmed (37 °C) PBS (Sigma-Aldrich) in a 2 mL microcentrifuge tube, followed by incubation at 37 °C for 15 min. Sperm were allowed to swim out for 10 min, after which 1 mL of the sperm suspension was collected from the far side of the dish.

### 2.6. Histopathological Assay

Testicular tissues fixed in Bouin’s solution (Sigma-Aldrich) were rinsed, dehydrated, embedded in paraffin (Sigma-Aldrich), sectioned (4 μm), mounted on slides, and stained with hematoxylin and eosin (H&E; Sigma-Aldrich). The slides were evaluated using a light microscope (MoticEasyScan Pro 6; Motic Asia, Hong Kong, China), by a single pathologist blinded to the experimental conditions. The injury score of testicular tissues was according to a previous report [12]. Each characteristic, including hemorrhage, edema, vascular congestion, polymorphonuclear leukocyte (PMN) infiltration, and germ cell ischemia, was scored on a scale from 0 to 3 (0, normal; 3, severe). The scores for hemorrhage, edema, vascular congestion, and PMN infiltration were assigned according to the affected area in the examined section (1, <20%; 2, 20–50%; 3, >50%). Overall testicular injury was further graded based on the total score, as previously described: 0–5, normal to mild injury; 6–10, moderate injury; and 11–15, severe injury [4].

### 2.7. Oxidative Stress Assay

Expression of HIF-1α, SOD-2 and MDA was assessed by immunohistochemistry and immunoblotting assays to assess oxidative stress in testicular tissues, as previously reported [4].

#### 2.7.1. Immunohistochemistry Assay

Immunohistochemistry assay was conducted following established protocols [12]. In brief, paraffin-embedded testicular sections (4 μm) were deparaffinized, rehydrated, and subjected to antigen retrieval in citrate buffer (pH 6.0; Sigma-Aldrich) at 95 °C for 20 min. Endogenous peroxidase activity was quenched with 3% hydrogen peroxide (Sigma-Aldrich) for 10 min, followed by blocking with 5% bovine serum albumin (BSA; Sigma-Aldrich) for 30 min at room temperature.

Sections were incubated overnight at 4 °C with primary antibodies against HIF-1α (1:200, IR113-466; iReal Biotechnology, Hsinchu City, Taiwan), SOD-2 (1:500, ab68155; Abcam, Cambridge, UK) or MDA (1:500, ab27642; Abcam). After washing, they were incubated with horseradish peroxidase (HRP)-conjugated secondary antibody (31460; Thermo Fisher Scientific, Waltham, MA, USA), developed with diaminobenzidine (DAB; Sigma-Aldrich), and counterstained with hematoxylin (Sigma-Aldrich). All sections were observed using a light microscope (MoticEasyScan Pro 6; Motic Asia) and analyzed using ImageJ version 1.52e (NIH, Bethesda, MD, USA).

#### 2.7.2. Immunoblotting Assay

Immunoblotting assay was conducted following established protocols [12]. In brief, frozen testicular samples were thawed and homogenized in radioimmunoprecipitation assay (RIPA) lysis buffer (Thermo Fisher) supplemented with a protease inhibitor cocktail (Thermo Fisher), and protein concentrations were measured using the bicinchoninic acid (BCA) assay kit (Thermo Fisher), according to the manufacturer’s instructions.

Equal amounts of protein (30 μg) were loaded to precast 10–12% Mini-PROTEAN^®^ TGX™ sodium dodecyl sulfate (SDS)–polyacrylamide gels (Bio-Rad Laboratories, Hercules, CA, USA) with a 4% stacking gel. Electrophoresis was performed in Tris–glycine–SDS running buffer (Thermo Fisher, Waltham, MA, USA) at 80 V through the stacking gel, then increased to 120 V for protein separation until the dye front reached the bottom of the gel. Proteins were then electrotransferred onto polyvinylidene difluoride (PVDF) membranes (0.45 µm pore size; Millipore, Burlington, MA, USA) using a wet transfer system in ice-cold transfer buffer (25 mM Tris, 192 mM glycine, 20% methanol; Thermo Fisher) at 100 V for 90 min at 4 °C.

Membranes were blocked with 5% non-fat dry milk in Tris-buffered saline containing 0.1% Tween-20 (TBST, Sigma-Aldrich) for 1 h at room temperature, then incubated overnight at 4 °C with primary antibodies against HIF-1α (1:1000, IR113-466; iReal Biotechnology), SOD-2 (1:2000, ab68155; Abcam), MDA (1:500, ab27642; Abcam) or glyceraldehyde 3-phosphate dehydrogenase (GAPDH, the internal control; 1:500, AF7021; Affinity Biosciences, Cincinnati, OH, USA). Following three washes in TBST (10 min each), membranes were incubated with anti-rabbit HRP-conjugated secondary antibody (1:5000, 31460; Thermo Fisher) for 1 h at room temperature. Bands were visualized using an enhanced chemiluminescence (ECL) detection kit (Thermo Fisher) and protein bands were visualized using the ChemiDoc MP Imaging System (Bio-Rad). Densitometric quantification was performed using an image processing software (VisionWorks^®^ Software, Version 8.20; Analytik Jena US, Upland, CA, USA).

### 2.8. Mitochondrial Complex I–V Activity in Testicular Tissue

The activities of mitochondrial respiratory chain complexes I–V in testicular tissue were evaluated using spectrophotometric assays with the corresponding Mitochondrial Complex I, II, III, IV, and V Activity Assay Kits (Cat. No. E-BC-K149-M, E-BC-K150-M, E-BC-K151-M, E-BC-K153-M; Elabscience, Houston, TX, USA). Briefly, approximately 100 mg of testis tissue was homogenized in the appropriate extraction buffer to obtain a 10% tissue homogenate, and mitochondria were isolated via differential centrifugation according to the kit instructions. An aliquot of the mitochondrial suspension was used for protein quantification, while the remainder was subjected to individual activity assays for each complex following the manufacturer’s protocols. For each assay, the mitochondrial fraction was incubated with the designated substrates, inhibitors, and cofactors specific to each complex, and absorbance changes were recorded at the specified wavelengths (e.g., 340 nm for complex I, 600 nm for complex II, 550 nm for complex III, 550 nm for complex IV, and 340 nm for complex V). Enzymatic activities were calculated according to the kit manuals and normalized to mitochondrial protein concentration to account for variations in sample loading.

### 2.9. Apoptosis Assay

Apoptosis in testicular tissues was assessed by using the terminal deoxynucleotidyl transferase dUTP nick end labeling (TUNEL) assay for DNA fragmentation and by immunoblotting for cleaved caspase-3, as previously described [4].

#### 2.9.1. TUNEL Assay

The TUNEL assay was performed using the In Situ Cell Death Detection Kit (Roche Diagnostics, Indianapolis, IN, USA) following the manufacturer’s protocol. In brief, paraffin-embedded testicular sections (4 µm) were processed as above described and then permeabilized with 0.1% Triton X-100 in 0.1% sodium citrate (both from Sigma-Aldrich) for 2 min on ice to facilitate reagent penetration.

Apoptotic cells were labeled by incubating sections with the TUNEL reaction mixture in a humidified chamber at 37 °C for 60 min in the dark. After rinsing in PBS, nuclei were counterstained with 4′,6-diamidino-2-phenylindole (DAPI; Sigma-Aldrich) for 5 min to visualize total cell nuclei. Sections were mounted and examined under a confocal microscope (LSM-780; Zeiss, Oberkochen, Germany), using appropriate excitation/emission filter settings for fluorescein (TUNEL) and DAPI. For quantification, five non-overlapping random fields (0.25 mm^2^ each) per section were imaged at ×400 magnification. The number of TUNEL-positive cells and total nuclei were counted using ImageJ software (NIH), and results were expressed as the mean number of TUNEL-positive cells per mm^2^ for each group.

#### 2.9.2. Immunoblotting Assay

For cleaved caspase-3 detection with immunoblotting, frozen testicular tissues were processed as above described. After electrophoresis and transfer to PVDF membranes, the membranes were incubated overnight at 4 °C with a primary antibody against cleaved caspase-3 (1:1000, ab2302; Abcam,) or GAPDH (1:500, AF7021; Affinity Biosciences), followed by incubation with an HRP-conjugated secondary antibody (1:5000, 31,460; Thermo Fisher). Immunoreactive bands were visualized using ECL and quantified by densitometric analysis, as above described.

### 2.10. Statistical Analysis

One-way analysis of variance (ANOVA) was used to assess differences between groups, followed by the Student–Newman–Keuls test for post hoc comparisons. All data were presented as the mean ± standard deviation. A significance level of *p* < 0.05 was considered statistically significant. Statistical analyses were performed using GraphPad Prism software version 10.1.0(316), 2023 (GraphPad Software, Inc., San Diego, CA, USA).

## 3. Results

### 3.1. PQQ Preserves Testicular Architecture, Sperm Count, and Testis-to-Body Weight Ratio Following T/D

**Figure 2:**
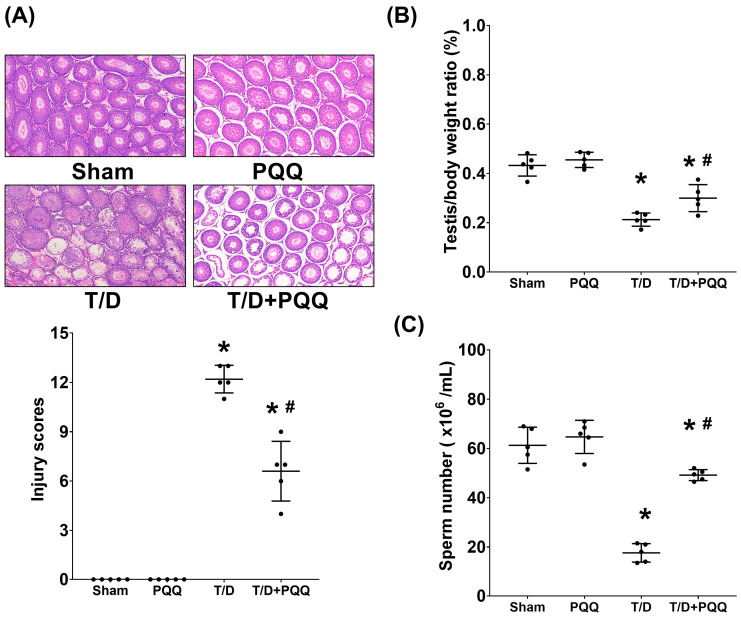
Effects of pyrroloquinoline quinone (PQQ) supplementation on mitigating testicular injury following torsion/detorsion (T/D). (**A**) Representative hematoxylin and eosin–stained sections of testicular tissues and injury scores from each group, using a light microscope (200×). (**B**) Quantification of testis/body weight ratio (%). (**C**) Sperm number. Data were obtained from 5 rats per group and are presented as mean ± standard deviation. Sham: sham surgery without treatment; PQQ: sham surgery with PQQ treatment; T/D: T/D surgery without treatment; T/D + PQQ: T/D surgery with PQQ treatment. * *p* < 0.05 vs. Sham; # *p* < 0.05, T/D + PQQ vs. T/D.

Histopathological analysis by H&E staining demonstrated that testicular T/D induced pronounced germinal epithelial disorganization, epithelial thinning, and luminal debris compared with Sham controls (Figure 2A). In contrast, rats in the T/D + PQQ group showed marked preservation of seminiferous tubule architecture, with fewer degenerative changes (Figure 2A). Quantitative injury scoring revealed significantly higher injury scores in the T/D group versus (vs.) Sham (*p* < 0.001), whereas PQQ treatment significantly reduced these scores (*p* < 0.001, T/D + PQQ vs. TD) (Figure 2A). Similarly, testis-to-body weight ratio, an indicator of testicular atrophy and functional decline, was also significantly reduced in T/D rats (*p* < 0.001, vs. Sham) but improved with PQQ administration (*p* = 0.017, T/D + PQQ vs. T/D) (Figure 2B). Moreover, sperm counts were markedly decreased in T/D compared with Sham (*p* < 0.001), and PQQ supplementation restored sperm numbers toward control levels (*p* < 0.001, T/D + PQQ vs. T/D) (Figure 2C). These findings indicate that TD induces structural and functional deterioration of the testes, which can be mitigated by PQQ therapy.

### 3.2. PQQ Reduces HIF-1α Upregulation in Testicular Tissues Following T/D

**Figure 3:**
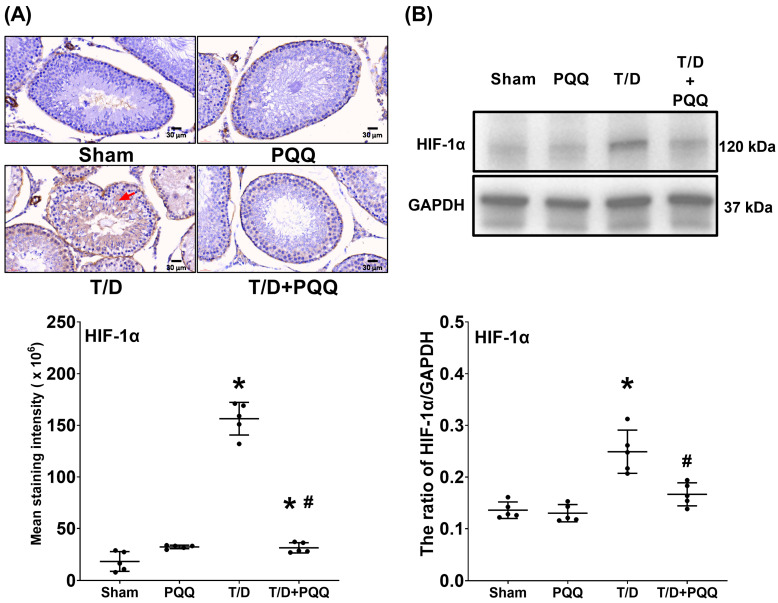
Effects of pyrroloquinoline quinone (PQQ) supplementation on attenuating hypoxia-inducible factor 1α (HIF-1α) upregulation in testicular tissues following torsion/detorsion (T/D). (**A**) Representative immunohistochemistry images of HIF-1α (indicated by red arrow) in testicular sections, with quantitative analysis of staining intensity across groups. (**B**) Representative Western blot analysis of HIF-1α protein expression, with GAPDH as an internal control, and corresponding quantitative densitometry. Data were obtained from 5 rats per group for immunohistochemistry and Western blotting and are presented as mean ± standard deviation. Sham: sham surgery without treatment; PQQ: sham surgery with PQQ treatment; T/D: T/D surgery without treatment; T/D + PQQ: T/D surgery with PQQ treatment. * *p* < 0.05 vs. Sham; # *p* < 0.05, T/D + PQQ vs. T/D.

Immunohistochemical staining (Figure 3A) demonstrated a pronounced elevation of hypoxia marker HIF-1α in the T/D group compared with Sham controls, reflecting the presence of aggravated hypoxic stress within the testicular microenvironment. Quantitative analysis of staining signals, together with Western blot validation (both *p* < 0.001, T/D vs. Sham; Figure 3A,B), consistently confirmed this observation, revealing a significant upregulation of HIF-1α in T/D testicular tissues. Notably, PQQ supplementation markedly attenuated the T/D-induced increase in HIF-1α expression, as the HIF-1α levels in the T/D + PQQ group were significantly lower than those in the T/D group (*p* < 0.001 and =0.001, T/D + PQQ vs. TD; Figure 3A,B). These findings provide robust evidence that PQQ effectively mitigates hypoxia induced by T/D, exerting protective effects at both the histological and molecular levels.

### 3.3. PQQ Restores SOD-2 Expression in Testicular Tissues Following T/D

**Figure 4:**
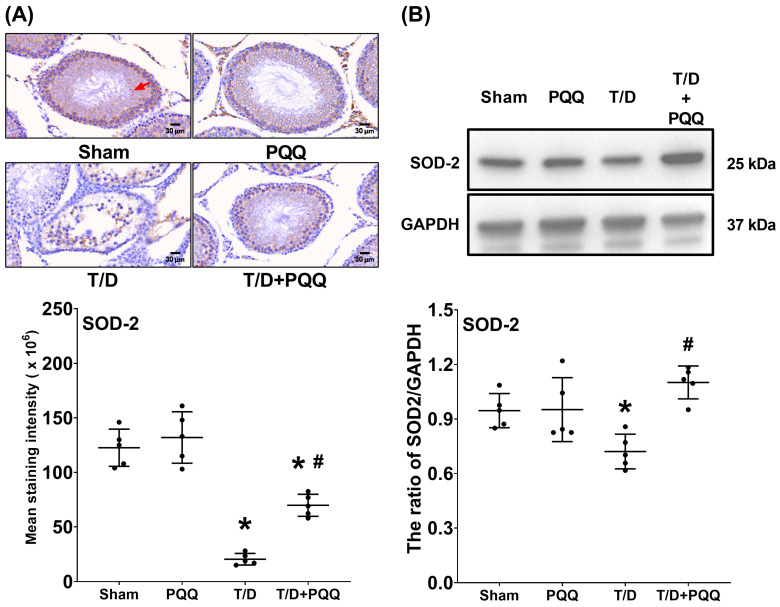
Effects of pyrroloquinoline quinone (PQQ) supplementation on mitigating superoxide dismutase 2 (SOD-2) downregulation in testicular tissues following torsion/detorsion (T/D). (**A**) Representative immunohistochemistry images of SOD-2 (indicated by red arrow) in testicular sections, with quantitative analysis of staining intensity across groups. (**B**) Representative Western blot analysis of SOD-2 protein expression, with GAPDH as an internal control, and corresponding quantitative densitometry. Data were obtained from 5 rats per group for immunohistochemistry and Western blotting and are presented as mean ± standard deviation. Sham: sham surgery without treatment; PQQ: sham surgery with PQQ treatment; T/D: T/D surgery without treatment; T/D + PQQ: T/D surgery with PQQ treatment. * *p* < 0.05 vs. Sham; # *p* < 0.05, T/D + PQQ vs. T/D.

Analysis of the antioxidant enzyme SOD-2 revealed a pronounced reduction in T/D testicular tissues relative to Sham controls, as demonstrated by immunohistochemical (IHC) staining (*p* < 0.001, T/D vs. Sham; Figure 4A). This loss of antioxidant capacity was corroborated by Western blotting and subsequent densitometric quantification (*p* = 0.040, T/D vs. Sham; Figure 4B), both of which consistently indicated significantly diminished SOD-2 expression in the T/D group. Importantly, PQQ supplementation effectively counteracted this decline, as the SOD-2 protein levels in the T/D + PQQ group were significantly higher than those in the T/D group (*p* = 0.001 and =0.001; Figure 4A,B). Collectively, these findings underscore the capacity of PQQ to reinforce antioxidant defenses under T/D-induced oxidative stress, thereby highlighting its potential role in preserving testicular redox homeostasis.

### 3.4. PQQ Reduces MDA Upregulation in Testicular Tissues Following T/D

**Figure 5:**
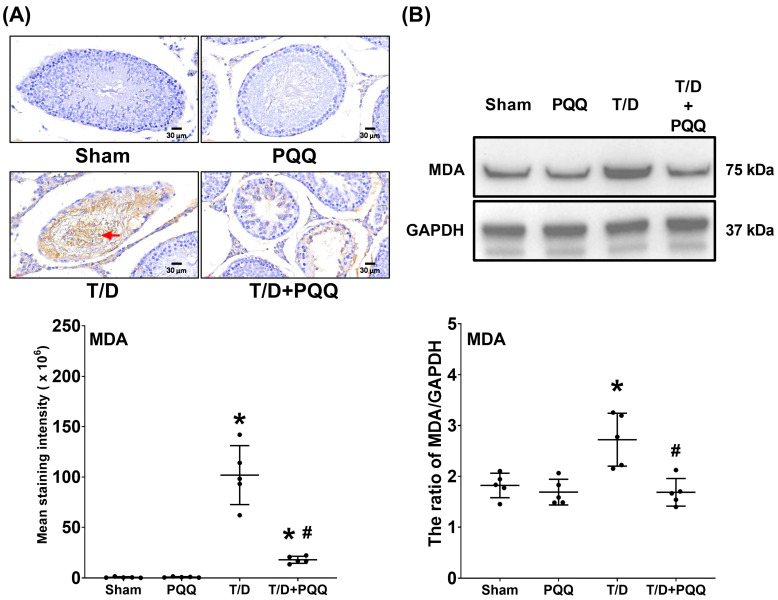
Effects of pyrroloquinoline quinone (PQQ) supplementation on attenuating malondialdehyde (MDA) upregulation in testicular tissues following torsion/detorsion (T/D). (**A**) Representative immunohistochemistry images of MDA (indicated by red arrow) in testicular sections, with quantitative analysis of staining intensity across groups. (**B**) Representative Western blot analysis of MDA protein expression, with GAPDH as an internal control, and corresponding quantitative densitometry. Data were obtained from 5 rats per group for immunohistochemistry and Western blotting and are presented as mean ± standard deviation. Sham: sham surgery without treatment; PQQ: sham surgery with PQQ treatment; T/D: T/D surgery without treatment; T/D + PQQ: T/D surgery with PQQ treatment. * *p* < 0.05 vs. Sham; # *p* < 0.05, T/D + PQQ vs. T/D.

Immunohistochemical staining and Western blotting assay for MDA, a classical biomarker of lipid peroxidation, demonstrated markedly elevated expression in the testicular tissues, reflecting enhanced oxidative injury to testes in the T/D group compared with Sham controls (*p* < 0.001 and =0.004; Figure 5A,B). In contrast, PQQ supplementation significantly attenuated MDA levels, as the MDA levels in the T/D + PQQ group were significantly lower than those in the T/D group (*p* < 0.001 and =0.001; Figure 5A,B). These findings indicate that PQQ effectively mitigates T/D-induced lipid peroxidation, thereby contributing to the preservation of testicular redox homeostasis and structural integrity.

### 3.5. PQQ Restores Mitochondrial OXPHOS Complex Activities in Testicular Tissues Following T/D

**Figure 6:**
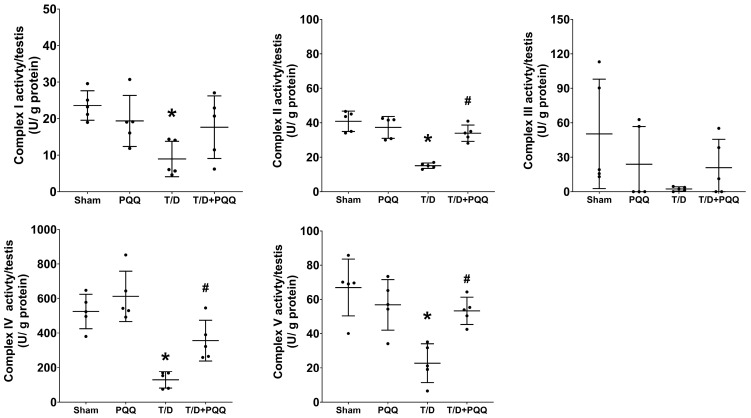
Effects of pyrroloquinoline quinone (PQQ) supplementation on restoring mitochondrial oxidative phosphorylation (OXPHOS) complexes in testicular tissues following torsion/detorsion (T/D). Data were obtained from 5 rats per group, and are presented as mean ± standard deviation. Sham: sham surgery without treatment; PQQ: sham surgery with PQQ treatment; T/D: T/D surgery without treatment; T/D + PQQ: T/D surgery with PQQ treatment. * *p* < 0.05 vs. Sham; # *p* < 0.05, T/D + PQQ vs. T/D.

Enzymatic activity assays of mitochondrial OXPHOS complexes revealed that T/D caused profound suppression of mitochondrial respiratory function, as indicated by significantly reduced activities of complexes I, II, IV, and V compared with Sham controls (*p* = 0.011, <0.001, <0.001, and <0.001, respectively; T/D vs. Sham) (Figure 6). This global impairment across the electron transport chain suggests substantial disruption of mitochondrial bioenergetics under T/D conditions. Remarkably, PQQ supplementation largely restored OXPHOS complex activities toward baseline levels, with significantly higher activities of complexes II, IV, and V observed in the T/D + PQQ group relative to the T/D group (*p* < 0.001, =0.022, and =0.001, respectively) (Figure 6). Although complex I activity showed a similar trend, the difference between the T/D + PQQ and T/D groups did not reach statistical significance (*p* = 0.178; Figure 6). Complex III activities did not differ significantly among groups (all *p* > 0.05), although a comparable trend was noted. Taken together, these findings indicate that PQQ exerts a broad-spectrum protective effect on mitochondrial OXPHOS function in testicular tissue following T/D.

### 3.6. PQQ Attenuates Apoptosis in Testicular Tissues Following T/D

**Figure 7:**
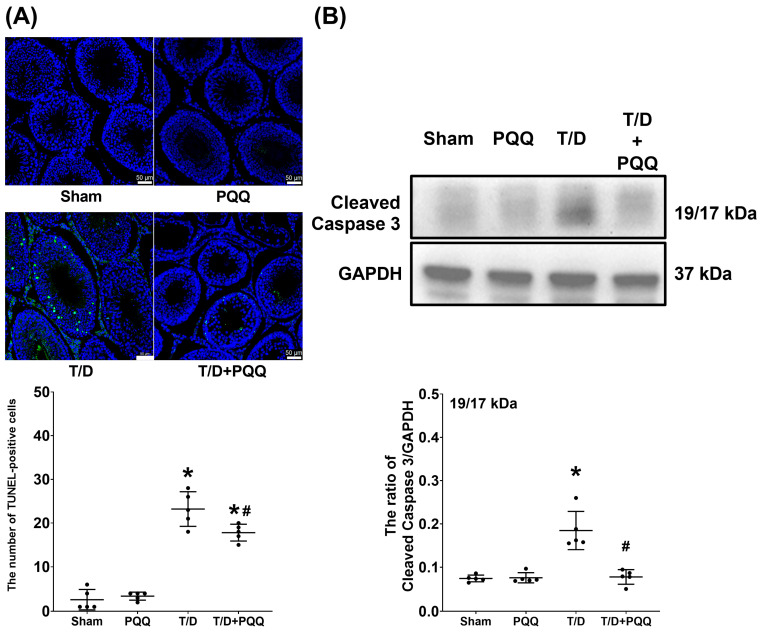
Effects of pyrroloquinoline quinone (PQQ) supplementation on restoring mitigating apoptosis in testicular tissues following torsion/detorsion (T/D). (**A**) Representative fluorescence images of terminal deoxynucleotidyl transferase dUTP nick end labeling (TUNEL) assay in testicular sections, showing TUNEL-positive nuclei indicated by green fluorescence, with quantification of TUNEL-positive cell number across groups. (**B**) Representative Western blot analysis of cleaved caspase-3 (19/17 kDa), with GAPDH as an internal control, and corresponding quantitative densitometry. Data were obtained from 5 rats per group for TUNEL assay and Western blotting and are presented as mean ± standard deviation. Sham: sham surgery without treatment; PQQ: sham surgery with PQQ treatment; T/D: T/D surgery without treatment; T/D + PQQ: T/D surgery with PQQ treatment. * *p* < 0.05 vs. Sham; # *p* < 0.05, T/D + PQQ vs. T/D.

The TUNEL assay revealed a marked increase in apoptotic signals and significantly higher numbers of TUNEL-positive cells in testicular tissues from the T/D group compared with Sham controls (*p* < 0.001; Figure 7A), indicating that T/D induces substantial testicular apoptosis. PQQ supplementation significantly reduced both apoptotic signals and TUNEL-positive cell counts (*p* = 0.018; T/D + PQQ vs. T/D; Figure 7A), thereby mitigating T/D-induced apoptotic injury. Consistently, Western blot analysis of cleaved caspase-3—a key executioner protease in the intrinsic apoptotic pathway—demonstrated markedly elevated expression in the T/D group relative to Sham (*p* < 0.001; Figure 7B). This upregulation was significantly suppressed by PQQ treatment (*p* < 0.001; T/D + PQQ vs. T/D; Figure 7B). Collectively, these findings provide strong evidence that PQQ confers anti-apoptotic protection in testicular tissue following T/D.

## 4. Discussion

This study provides novel evidence that oral PQQ supplementation confers robust protection against T/D-induced ischemia–reperfusion injury in the testis. The protective effects of PQQ were demonstrated at multiple levels, including preservation of testicular architecture, restoration of mitochondrial OXPHOS complex function, reinforcement of antioxidant defenses, attenuation of oxidation, and suppression of apoptosis. To our knowledge, this is the first study to delineate the integrated protective mechanisms of PQQ in T/D injury, underscoring its translational potential in reproductive medicine. As PQQ administration in this study was initiated after surgical detorsion, the results specifically support its role as a post-operative supplemental therapy aimed at mitigating reperfusion-associated oxidative and mitochondrial damage. Such intervention could potentially improve testicular recovery and functional preservation following surgery. Importantly, PQQ was selected for evaluation based on prior toxicological evidence showing that daily oral doses up to 400 mg/kg body weight produced no significant adverse effects in rats [22]. Consistent with these findings, our analyses revealed no significant differences in serum biochemical markers of hepatic and renal function—including aspartate aminotransferase (AST), alanine aminotransferase (ALT), blood urea nitrogen (BUN), and creatinine—among experimental groups (Supplementary Figure S1), further supporting the safety and tolerability of PQQ supplementation under the present experimental conditions.

Histopathological analysis revealed extensive disorganization of seminiferous tubules, epithelial thinning, and luminal debris in T/D testes, findings that are consistent with prior reports showing that ischemia–reperfusion injury disrupts the germinal epithelium and compromises spermatogenesis [7,8,12]. These structural derangements were paralleled by functional impairment, evidenced by reductions in both sperm count and testis-to-body weight ratio—established markers of testicular injury. Importantly, PQQ supplementation significantly mitigated these changes, supporting its role as a candidate therapeutic intervention capable of preserving both morphology and reproductive capacity.

At the molecular level, T/D markedly increased HIF-1α, reflecting exacerbated tissue hypoxia. In parallel, SOD-2, a critical mitochondrial antioxidant enzyme, was suppressed, while MDA, an index of lipid peroxidation, was elevated. These alterations reflect a collapse of redox homeostasis, in line with previous findings that oxidative stress is a dominant driver of T/D-induced testicular injury [8,16]. Recent studies further support that excessive ROS generation disrupts mitochondrial membrane integrity, impairs spermatogenic cell metabolism, and triggers lipid peroxidation-driven ferroptotic and apoptotic pathways in the testes [23]. PQQ treatment counteracted these changes by reducing HIF-1α and MDA while restoring SOD-2, indicating that it alleviates hypoxia-driven stress and strengthens endogenous antioxidant defenses. These effects may relate to PQQ’s known ability to stimulate mitochondrial biogenesis and activate antioxidant signaling cascades such as PGC-1α and cAMP response element-binding protein (CREB) pathways [16,24].

Mitochondrial dysfunction is a hallmark of T/D pathology. In agreement with our prior proteomic findings of downregulated electron transport chain subunits, including NADH dehydrogenase [ubiquinone] iron-sulfur protein 1 (NDUFS1), succinate dehydrogenase complex subunit C (SDHC), ATP synthase F0 subunit J and subunit I (ATP5J and ATP5CI, respectively) [12], the present study confirmed profound suppression of OXPHOS complex activities. This global impairment indicates diminished mitochondrial bioenergetics and ATP production, which are essential for spermatogenesis and sperm motility [11]. PQQ supplementation largely restored OXPHOS activity, supporting the conclusion that PQQ rescues mitochondrial respiratory function. By restoring mitochondrial respiratory function, PQQ likely supports the high energetic demands of spermatogenesis and contributes to the functional recovery of injured testes. These findings align with recent work indicating that antioxidant interventions restoring mitochondrial redox balance can mitigate oxidative sperm damage and preserve fertility [25].

Apoptosis also emerged as a major contributor to T/D-induced injury. We observed a pronounced increase in DNA fragmentation, TUNEL-positive cells, and cleaved caspase-3 expression, consistent with previous evidence linking oxidative stress to activation of the intrinsic apoptotic pathway [8]. PQQ significantly suppressed these pro-apoptotic responses, confirming its capacity to protect testicular tissues from apoptosis. These findings are in line with prior studies demonstrating the anti-apoptotic actions of PQQ in other ischemia–reperfusion injury models, such as cerebral and hepatic injury [26,27], and reinforce the concept that PQQ exerts protection through convergent, mitochondria-centered mechanisms.

**Figure 8:**
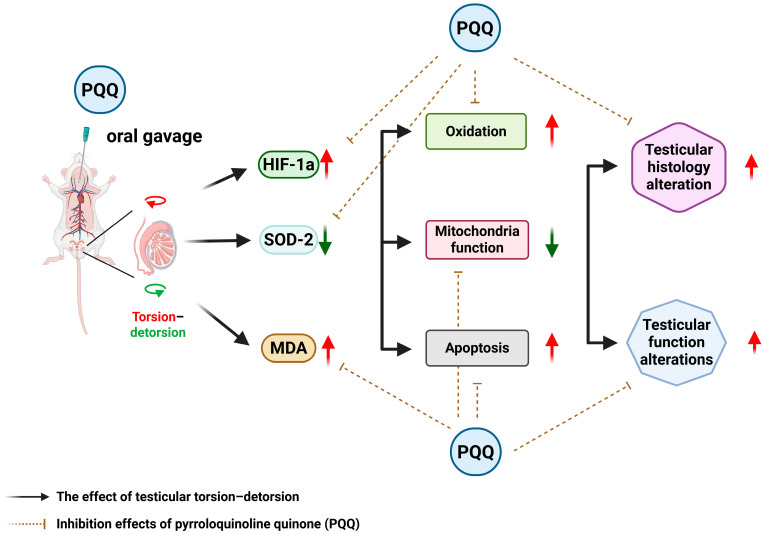
The diagram summarizing the effects and mechanisms of pyrroloquinoline quinone (PQQ) supplementation on attenuating testicular torsion-detorsion (T/D) induced testicular injury in rats. HIF-1α: hypoxia-inducible factor 1α; SOD-2: Superoxide dismutase 2; MDA: malondialdehyde.

Taken together, our findings demonstrate that PQQ provides broad-spectrum protection against T/D-induced ischemia–reperfusion injury by alleviating hypoxia, enhancing antioxidant capacity, reducing lipid peroxidation, rescuing mitochondrial OXPHOS function, and inhibiting apoptosis. By targeting mitochondria-centered oxidative and apoptotic cascades, PQQ not only prevents redox imbalance but may also counteract emerging forms of oxidative cell death such as ferroptosis. This constellation of effects highlights PQQ as a promising therapeutic candidate for testicular torsion–detorsion and potentially for other reproductive disorders characterized by oxidative stress and mitochondrial dysfunction. The effects and mechanisms of PQQ supplementation on attenuating T/D-induced testicular injury in rats are summarized in Figure 8.

In addition to the intrinsic mechanisms of oxidative damage caused by T/D-induced ischemia–reperfusion injury, environmental pollutants may serve as important co-factors that exacerbate testicular injury. Epidemiological and experimental studies have shown that exposure to heavy metals and endocrine-disrupting chemicals can impair male reproductive health by inducing oxidative stress, mitochondrial dysfunction, and apoptosis—pathways that closely parallel those observed in T/D-induced ischemia–reperfusion injury [28]. Pollutants such as mercury, cadmium, and lead promote excessive reactive oxygen species generation, disrupt mitochondrial bioenergetics, and trigger germ cell apoptosis, thereby lowering the threshold for ischemic damage [29]. Moreover, pollutants can interfere with the interaction between sperm nuclear basic proteins and DNA, compromising chromatin stability and increasing susceptibility to oxidative DNA damage [28,29]. These convergent mechanisms suggest that environmental exposures may amplify I/R-induced testicular injury. In this context, PQQ, with its potent antioxidant and mitochondria-protective properties, may also mitigate pollution-related oxidative stress and mitochondrial impairment. The ability of PQQ to enhance mitochondrial biogenesis, preserve redox balance, and suppress apoptosis highlights its potential not only as a therapeutic agent for testicular I/R injury but also as a broader protective strategy against environmental pollutant-induced reproductive toxicity.

Moreover, beyond its well-established antioxidant and mitochondrial-protective actions, PQQ may exert regulatory effects through epigenetic mechanisms. Bauerly et al. [30] reported that PQQ deprivation altered histone H3 lysine methylation patterns in genomic regions associated with neuroprotective genes and retrotransposon sequences, thereby influencing gene transcription involved in mitochondrial biogenesis and stress responses. These observations suggest that PQQ can modulate chromatin structure and transcriptional activity, potentially contributing to its broader cytoprotective functions. Nevertheless, as these effects have been characterized mainly in neuronal systems, further studies are required to elucidate whether PQQ induces similar epigenetic modifications in the testis and to clarify their relevance in the context of ischemia–reperfusion injury.

Emerging evidence indicates that adherence to the Mediterranean diet—a dietary pattern rich in fruits, vegetables, whole grains, olive oil, fish, and nuts—confers protective effects on male reproductive health [31]. The high content of antioxidants, vitamins, and omega-3 fatty acids in this diet helps reduce systemic oxidative stress and inflammation, key mechanisms underlying T/D-induced ischemia–reperfusion injury. Several studies have reported improved sperm motility, concentration, and morphology among men following this dietary pattern, likely through enhanced mitochondrial function and protection against lipid peroxidation [31,32]. These mechanisms parallel the antioxidant and mitochondrial benefits observed with PQQ supplementation, suggesting potential synergistic or complementary effects. Together, these findings underscore that both nutritional interventions and targeted antioxidant therapies such as PQQ may offer promising strategies for mitigating oxidative testicular injury and male infertility.

Oxidative stress is a major contributor to male infertility, and antioxidant supplementation has been widely explored as a therapeutic strategy to improve sperm quality and reproductive outcomes. Beneficial effects of antioxidants such as vitamins C and E, coenzyme Q10, resveratrol, and PQQ include the reduction in ROS, stabilization of mitochondrial function, and prevention of lipid peroxidation and DNA fragmentation in spermatozoa [25]. In this study, as above-mentioned, PQQ treatment significantly reduced oxidative stress markers, restored mitochondrial respiratory enzyme activity, and suppressed apoptosis in the testis, confirming its beneficial antioxidant and mitochondria-protective effects in vivo. However, the use of antioxidants is not without limitations. Excessive or unregulated supplementation may disrupt physiological redox signaling essential for sperm maturation, capacitation, and acrosome reaction, resulting in the so-called “antioxidant paradox” [33]. Moreover, heterogeneity in dosing regimens and patient selection has led to inconsistent clinical outcomes, and some studies have reported mild adverse effects such as nausea or dyspepsia associated with long-term antioxidant use [34]. Thus, while PQQ demonstrated robust antioxidant efficacy in the present study, the optimal application of antioxidants in male fertility management should be guided by mechanistic understanding, individualized dosing, and rigorous clinical validation to balance efficacy and safety.

Nevertheless, several limitations warrant consideration. First, the rat T/D model, though widely used for mechanistic studies, does not fully recapitulate the anatomical and physiological complexity of human testicular torsion. Second, only a single dose and treatment schedule of PQQ were tested, leaving dose–response relationships, therapeutic windows, and long-term fertility outcomes to be explored. Third, the sample size for mitochondrial enzymatic assays was relatively small, and the data showed inter-individual variability, which may limit statistical power. Although ATP production was not directly assessed, the observed consistent trends across complexes I–V likely reflect proportional changes in overall mitochondrial bioenergetic capacity. Future studies with larger sample sizes and direct ATP production assays are warranted to validate these findings. Fourth, only cleaved caspase-3 protein levels were measured, without normalization to total caspase-3, which may limit the precision of apoptotic quantification; subsequent studies should include both total and cleaved forms for comprehensive evaluation. Fifth, the lack of significant change in SOD-2 expression between the Sham and PQQ groups (Figure 4) suggests that PQQ’s antioxidant effects may operate primarily through enhancement of mitochondrial redox balance and modulation of upstream regulators such as PGC-1α, rather than direct induction of antioxidant enzymes; time-dependent and transcriptional regulation of mitochondrial antioxidants should be explored in future work. Sixth, the mechanistic insights remain correlative; future work should directly interrogate pathways such as PGC-1α activation or CREB phosphorylation using genetic or pharmacological approaches to confirm causality. Seventh, the study did not include direct comparisons between PQQ and other antioxidant agents. Although the experiments were aligned with the molecular framework of oxidative and apoptotic signaling in the T/D model, future studies should perform head-to-head evaluations with established antioxidants (e.g., coenzyme Q10, resveratrol, or N-acetylcysteine) or target-specific interventions to determine whether PQQ provides distinct or superior protection against testicular ischemia–reperfusion injury. Eighth, this study was conducted exclusively in male rats, and potential sex-related differences in the effects of PQQ were not evaluated. As ischemia–reperfusion injury can also occur in female reproductive organs, such as ovarian torsion or uterine ischemia, and given that sex hormones and mitochondrial function may influence redox homeostasis [35], further investigations are warranted to determine whether PQQ exerts similar or distinct protective mechanisms in females. Ninth, this study did not directly assess sperm functional parameters such as motility, capacitation, acrosome reaction, or sperm-specific apoptosis. Apoptosis analysis was limited to seminiferous tubules, while sperm counts—obtained from motile sperm that swam out within 10 min—only indirectly reflected viable sperm populations. Although these findings suggest improved sperm quality after PQQ treatment, detailed evaluation of sperm function and apoptosis remains warranted in future studies. Finally, translation to clinical practice will require comprehensive evaluation of PQQ pharmacokinetics, safety, and efficacy in large animal models and rigorously designed clinical trials.

## 5. Conclusions

In summary, this study provides novel evidence that pyrroloquinoline quinone (PQQ) supplementation exerts robust protection against testicular torsion–detorsion (T/D)-induced ischemia–reperfusion injury. PQQ preserved seminiferous tubule architecture, maintained sperm count and testis-to-body weight ratio, and attenuated structural deterioration. At the molecular level, PQQ reduced hypoxia-inducible factor-1α (HIF-1α), restored mitochondrial superoxide dismutase 2 (SOD-2), and decreased malondialdehyde (MDA) accumulation, thereby reinforcing antioxidant defenses and mitigating lipid peroxidation. Moreover, PQQ rescued mitochondrial oxidative phosphorylation (OXPHOS) complex activities and suppressed testicular apoptosis. Collectively, these findings demonstrate that PQQ acts through integrated anti-hypoxic, antioxidant, mitochondrial-preserving, and anti-apoptotic mechanisms to safeguard testicular function. While further studies are warranted to validate the mechanistic pathways and optimize dosing strategies, these results highlight PQQ as a promising therapeutic candidate for testicular T/D and potentially other reproductive pathologies driven by oxidative stress and mitochondrial dysfunction.

## Data Availability

All data supporting the findings of this study are contained within the article. No additional data are available.

## Supplementary Materials

The following supporting information can be downloaded at: https://www.mdpi.com/article/10.3390/antiox14111312/s1, Supplementary Figure S1: Serum biochemical analysis. Supplementary Figure S2: Uncropped Western blotting gel images.

## Abbreviations

The following abbreviations are used in this manuscript:

T/D	Testicular torsion–detorsion
IRI	Ischemia–reperfusion injury
PQQ	Pyrroloquinoline quinone
OXPHOS	Oxidative phosphorylation
HIF-1α	Hypoxia-inducible factor-1α
ROS	Reactive oxygen species
MDA	Malondialdehyde
SOD-2	Superoxide dismutase 2
PBS	Phosphate-buffered saline
H&E	Hematoxylin and eosin
PMN	Polymorphonuclear leukocyte
SDS	Sodium dodecyl sulfate
PVDF	Polyvinylidene difluorid
TBST	Tris-buffered saline containing 0.1% Tween-20
GAPDH	Glyceraldehyde 3-phosphate dehydrogenase
HRP	Horseradish peroxidase
TUNEL	Terminal deoxynucleotidyl transferase dUTP nick end labeling
DAPI	4′,6-diamidino-2-phenylindole
NIH	National Institutes of Health
ECL	Enhanced chemiluminescence
ANOVA	One-way analysis of variance
PGC-1α	Peroxisome proliferator-activated receptor gamma coactivator 1α
CREB	cAMP response element-binding protein
NDUFS1	NADH dehydrogenase [ubiquinone] iron-sulfur protein 1
SDHC	Succinate dehydrogenase complex subunit C
ATP5J	ATP synthase F0 subunit J
ATP5CI	ATP synthase F0 subunit I
