# Supplementary figures and images for: Pyrroloquinoline Quinone Mitigates Testicular Injury and Reduces Oxidative Stress, Mitochondrial Dysfunction, and Apoptosis in Rats with Testicular Ischemia–Reperfusion Injury
Source: Antioxidants (Basel). 2025 Oct 31;14(11):1312. doi: 10.3390/antiox14111312 (PMC12649516; doi:10.3390/antiox14111312)

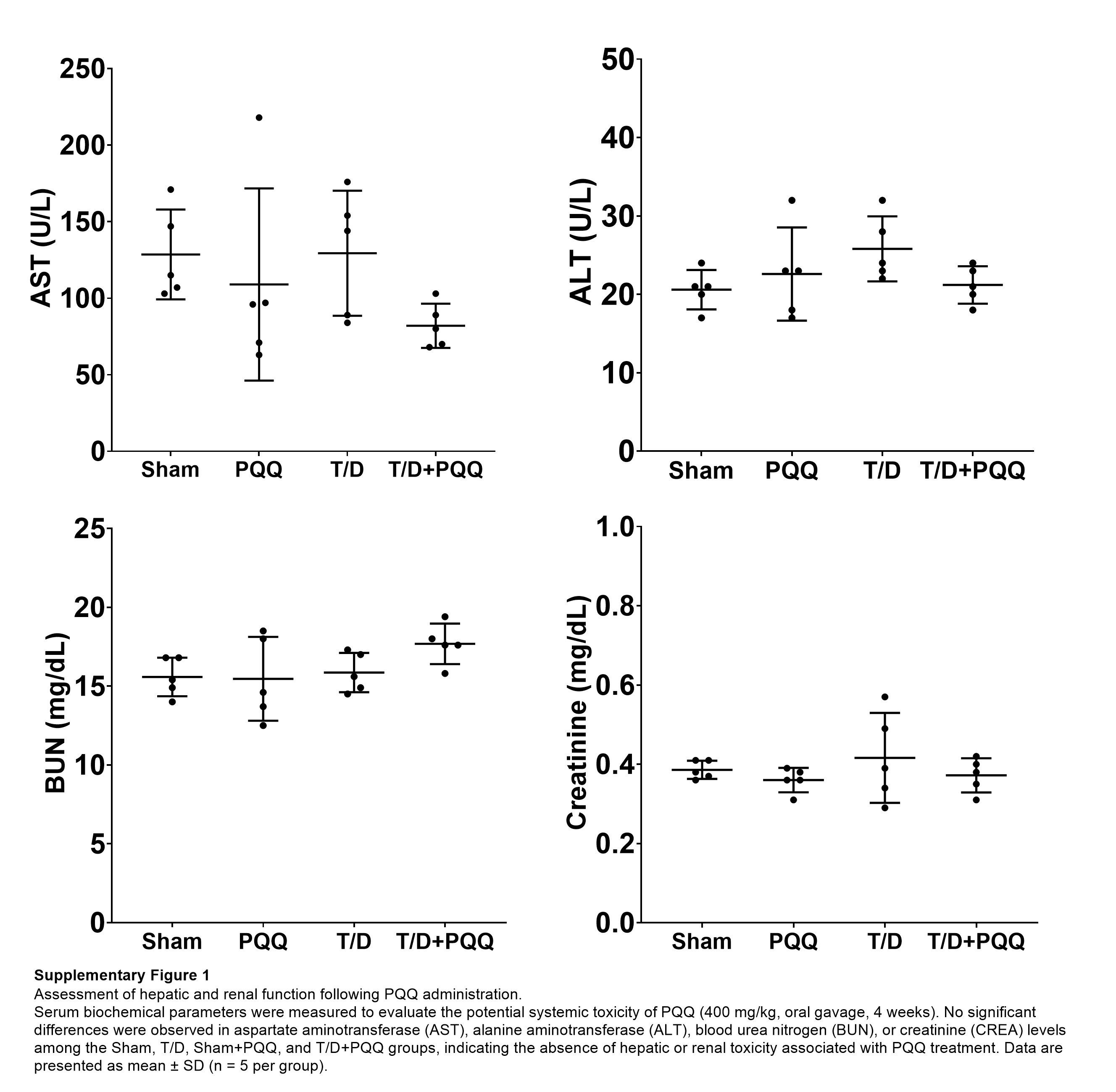

Supplement: Supplementary file 1 [file antioxidants-14-01312-s001.zip › Supplemental figure S1.tif]

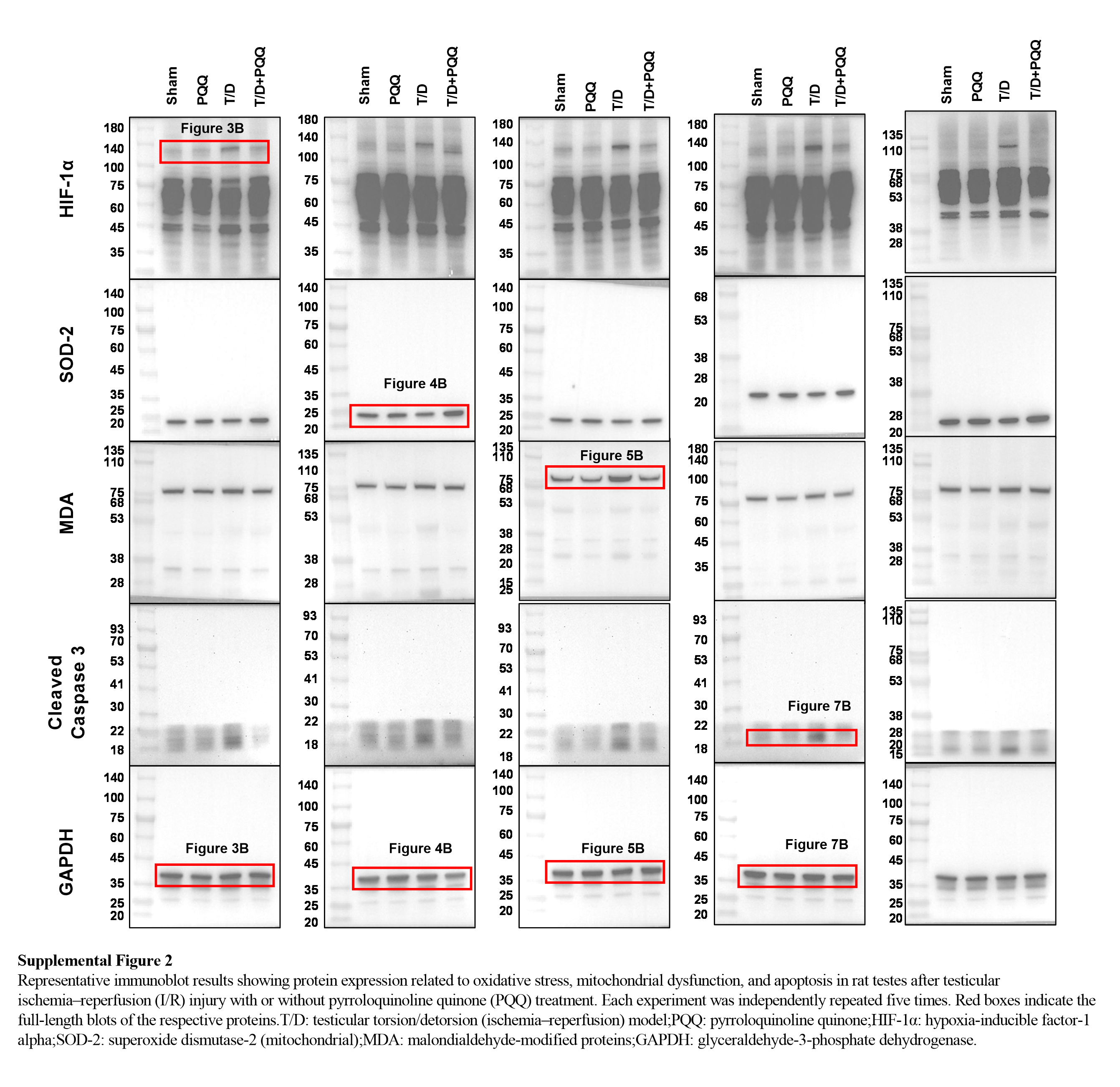

Supplement: Supplementary file 1 [file antioxidants-14-01312-s001.zip › Supplemental figure S2.tif]
